# Balance Training With Vibrotactile Neurofeedback and *Ginkgo Biloba* Extract in Age-Related Vertigo

**DOI:** 10.3389/fneur.2021.691917

**Published:** 2021-11-30

**Authors:** Lars Decker, Dietmar Basta, Martin Burkart, Arne Ernst

**Affiliations:** ^1^Department of Otolaryngology, Unfallkrankenhaus Berlin, Hospital of the University of Berlin, Charité Medical School, Berlin, Germany; ^2^Dr. Willmar Schwabe GmbH & Co. KG, Karlsruhe, Germany

**Keywords:** presbyvestibulopathy, balance training, vibrotactile neurofeedback, *Ginkgo biloba*, balance deficit

## Abstract

**Background:** Balance training with vibrotactile neurofeedback (VNF) can improve balance and subjective impairment in age-related vertigo and dizziness. *Ginkgo biloba* dry extract EGb 761 has been shown to improve subjective impairment in chronic vertigo and the efficacy of conventional balance training. The combination was expected to work synergistically in this difficult-to-treat population.

**Objectives:** To demonstrate the efficacy of VNF added to EGb 761 for age-related vertigo and dizziness.

**Design:** Multicenter, prospective, controlled, randomized, single-blind, two-arm trial (German Clinical Trials Register https://www.drks.de No. DRKS00007633).

**Setting:** Specialist offices and tertiary care outpatient department.

**Participants:** One hundred and twenty subjects aged 60+ years with chronic dizziness for over 3 months, a Dizziness Handicap Inventory (DHI) Sum Score >25 and fall risk in balance-related situations as measured by the geriatric Standard Balance Deficit Test Composite Score (gSBDT-CS)>40. Patients with other distinct vestibular pathology (e.g., Meniére's disease, stroke, BPPV) were excluded.

**Intervention:** EGb 761 (80 mg twice daily for 12 weeks) plus 10 days of individually adapted balance training with VNF, randomized 1:1 to sensitive (active) or non-sensitive (sham) neurofeedback.

**Measurements:** The change in gSBDT-CS after 6 weeks (primary), other gSBDT outcomes, DHI, cognition, hearing, and safety.

**Results:** One hundred nine of 120 enrolled subjects received both treatments at least once. Over 12 weeks, the gSBDT-CS improved by 6.7 (active) vs. 4.5 (sham). There was a difference in favor of the active treatment of −2.4 (95% CI −5.4; 0.6) after 6 weeks. Under active treatment, more pronounced effects occurred in all secondary analyses and in nearly all secondary endpoints. The DHI sum score decreased from 44.1 to 31.1 in the total sample with a treatment group difference after 6 weeks of −3.1, 95% CI (−7.1; 0.9). No safety issues were reported.

**Conclusion:** Over 12 weeks, the combination of balance training with VNF and *Ginkgo biloba* dry extract EGb 761 reached a clinically relevant improvement of age-related vertigo and dizziness with a good pharmacological safety profile.

## Introduction

Age-related vertigo and dizziness is defined as a chronic vestibular syndrome characterized by unsteadiness, gait disturbance, and/or recurrent falls in the presence of mild bilateral vestibular deficits (1). It is characterized by a complex loss in spatio-temporal integration as induced by degeneration of vestibular sensory cells within the semicircular canals, followed by the macular organs (2), along with other age-related deficits of vision, proprioception, cortical, cerebellar and/or extrapyramidal function, muscle mass and other medical conditions (e.g., cardiovascular disease, neurovascular disorders). According to national and international guidelines balance training (vestibular rehabilitation) is a key treatment component, but with limited effectiveness (3–6). Due to the significant burden of the condition (7) and poor response to balance training alone (8), enhanced training approaches have been developed. Vibrotactile neurofeedback (VNF) improves the effect of conventional balance training (9, 10). While performing VNF, patients receive a non-painful, vibrotactile vibration pulse during balance training exercises if they exceed certain limits of postural stability recorded as body sway at the center of body gravity (at about the hip circumference). Although the mechanism of action of VNF has not been fully understood, it is assumed that the effect is mediated by motor learning processes based on the principles of classical and operand conditioning (9). Data on VNF in patients with age-related vertigo and dizziness are available, but so far with limited patient numbers (9–11).

EGb 761, a special extract from dried leaves of *Ginkgo biloba L*., has been approved for the treatment of vertigo of vascular and involutional origin in several countries. Placebo-controlled trials have shown that EGb 761 improves subjective impairment in patients with chronic vertigo and enhances the efficacy of conventional balance training (12, 13). This might result from enhanced classic and operand conditioning observed in animal experiments (14). Recently, two meta-analyses supported the use of EGb 761 for dizziness in the elderly: Tan et al. (15) looked into the systemic effects and side effects of EGb 761 in the treatment of mild cognitive impairment and dementia and reported that the incidence of dizziness, particularly in Alzheimer's disease, was significantly reduced as compared to placebo. A meta-analysis (16) of EGb 761 effects in elderly patients with dementia provided evidence for an improvement in stance and gait.

It has been a long-standing demand to perform more controlled clinical trials in the management of the elderly with chronic dizziness (17, 18). The present multicenter study was aimed at investigating whether the combination of both treatment modalities (VNF plus EGb 761) can improve this condition in this difficult-to-treat population.

## Materials and Methods

This study was designed as a confirmatory, multicenter, prospective, controlled, randomized, single-blind, two-arm, parallel group adaptive clinical trial (Eudra-CT No: 2014-000303-28, EUDAMED No: CIV-14-08-012482, German Clinical Trials Register No: DRKS00007633).

Regulatory approvals were obtained according to German law for drugs and medical devices. Also, favorable opinions of all related Ethic committees were obtained prior to patient enrollment. The study was conducted in four centers in Germany between January 2015 and June 2018: A tertiary care outpatient unit (Department of Otolaryngology, Unfallkrankenhaus Berlin, Hospital of the University of Berlin), two professional clinical trial organizations (Medizinisches Studienzentrum, Würzburg and Synexus Clinical Research GmbH, Leipzig), and an ENT outpatient practice in Heidelberg. The sites recruited 56, 30, 9, and 25 patients.

### Patients and Treatment Arms

Main inclusion criteria were subjects aged 60+ years with chronic dizziness for over three months (i.e., imbalance and subjective gait disturbance), a Dizziness Handicap Inventory (DHI) sum score >25 and a geriatric Standard Balance Deficit Test (gSBDT) composite score >40. Patients with other distinct vestibular pathology (e.g., Meniére's disease, stroke, BPPV) were excluded. The patients were examined clinically, the history was taken (incl. DHI) and an otoscopy was performed. The neurotological workup included a Dix-Hallpike-maneuver, the assessment of the subjective haptic vertical, pure-tone audiograms (PTA), and a mobile posturography by performing the geriatric Standard Balance Test (gSBDT) (19). In addition, an ECG, the trail-making test (TMT) and standard laboratory tests were done.

The study consisted of a screening period (up to 14 days) and an 80–88 days' treatment period with five regular clinical trial visits.

After having obtained the written, informed consent and following screening, the patients received 12 weeks of open label treatment with EGb 761 (80 mg orally, twice daily). EGb 761^®^ (active ingredient of Tebonin^®^, Schwabe Pharmaceuticals, Karlsruhe, Germany) is a quantified dry extract from dried leaves of *Ginkgo biloba L*. (Ginkgoaceae; maidenhair tree), drug-extract-ratio 35–67:1, primary extraction solvent: acetone 60% (w/w). The extract is adjusted to 22.0–27.0% ginkgo flavonoids, calculated as ginkgo flavonol glycosides and 5.0–7.0% terpene lactones consisting of 2.8–3.4% ginkgolides A, B, C and 2.6–3.2% bilobalide and contains <5 ppm ginkgolic acids. EGb 761 is manufactured according to GMP (Good Manufacturing Practice) and GACP (Good Agricultural and Collection Practice for Starting Materials of Herbal Origin) standards of the European Medicines Agency and is approved as an herbal drug in numerous European, Asian and Latin-American countries. EGb 761 was used as background treatment for all patients and not randomized, because it is an approved treatment modality for decades and efficacy vs. placebo has been established (12, 13) for this drug.

After 4 weeks, patients received additional individually adapted balance training for 10 days with (depending on randomization) active or inactive (sham) VNF using the training system VertiGuard^®^ RT (Zeisberg GmbH, Metzingen, Germany). Briefly, the medical device fixed to a hip belt registers the body movements in the sagittal and transversal levels (roll and pitch) during standardized balance exercises and automatically matches the measured values with age- and gender-specific normal data. The VNF training was performed by using the six poorest (in outcome) exercises from the diagnostic run of the gSBDT at the baseline visit. The gSBDT comprises a set of 14 different standardized everyday life stance and gait conditions, such as standing with eyes open or closed, walking with and without head movements, or standing up (20). The training was carried out for 30 min on-site daily for ten consecutive working days. During the training session, the patient performed the selected exercises with five repetitions. The belt reacts to excessive body movements with vibration. For more details of the VNF training see (9). For sensitive neurofeedback, the feedback thresholds were adapted daily by the study nurse, so that patients received optimal neurofeedback, while for sham feedback, the threshold was always kept at maximum and the stimulus was provided randomly. Single blinding of neurofeedback was chosen so that the patients did not know if they had been assigned to balance training with sensitive (active) or insensitive (sham) neurofeedback. A double-blind design could not be realized, as this would have required manipulation of the medical device software which was technically not feasible.

### Allocation of Patients and Statistical Analysis/Outcome Measures

Allocation concealment was achieved by consecutively numbered sealed randomization envelopes produced by a data manager not involved in the trial conduct. The randomization list was generated with Research Ramdomizer (21), block size (size = 4) was not disclosed to the investigators. After enrolment of an individual patient, the investigator allocated the patient to the treatment indicated in the envelope with the next patient number.

The primary outcome variable was the change in fall risk (gSBDT composite score) after a total of 6 weeks of treatment, with 4 weeks on EGb 761 alone and 14 days on EGb 761 plus VNF (10 training days). Secondary endpoints included gSBDT composite score changes after 4 and 12 weeks of treatment, gSBDT components, proportion of patients with a fall risk >50%, DHI sum and subscores, hearing ability, cognitive performance as well as safety and tolerability.

The subjective impairment due to dizziness was measured using the Dizziness Handicap Inventory (22). The sum score of the given answers reflects the degree of everyday impairment of the patient due to balance disorders. In addition, the subscores of the DHI for functional, physical and emotional stress were evaluated.

Cognitive performance was assessed by the Trail Making Test (23). Hearing impairment is a clinically useful indicator of increased fall risk (24, 25), so that a PTA was done pre/post intervention. Hearing thresholds were averaged for both ears and frequencies assessed (0.5, 1, 2, 4, and 8 kHz).

The sample size was calculated according to Friede and Kieser (26) and was based on assumptions from previous studies (9, 10). Assuming a clinically relevant difference of Δ = 4 points in the gSBDT-CS and a standard deviation of σ = 9.9, for a two-sided significance level of α = 0.05 a total sample size of *n* = 62 patients was required to achieve a power of 1-β = 0.80 with an Analysis of Covariance (ANCOVA) using baseline values as covariates. Due to uncertainties in these assumptions, the trial was planned as group sequential design. After availability of primary efficacy data of 50 patients, the sample size was recalculated based on the observed data for the primary endpoint variance and correlation with baseline values of the total sample without unblinding the trial. The initial sample size of 62 patients was then adjusted to the a priori defined maximum total number of 120 patients.

The primary endpoint was analyzed with a mixed model with repeated measurements (MMRM), where treatment is included into the model as fixed factor and baseline value as covariate, each with a visit interaction term. Sensitivity analyses were Complete Case, Last Observation Carried Forward and Multiple Imputation analyses. Descriptive *p*-values of appropriate statistical tests and associated 95% confidence intervals were calculated for secondary endpoints.

## Results

One hundred and ninety patients were screened, and 120 randomized, 59 patients to EGb 761 plus active VNF and 61 patients to EGb 761 plus sham VNF. Most frequent reasons for screening failure were DHI ≤ 25 (*n* = 25), GSBDT ≤ 40% (*n* = 12), and BPPV (*n* = 11). Various medical (e.g., diseases affecting resorption, intake of a *Ginkgo Biloba* products within the last 12 weeks, severe or acute general diseases within the last 4 weeks, severe or instable internal disorders, hypersensitivity against one of the ingredients of the study drug, substance misuse or addiction current or in medical history), administrative or ethical reasons precluded randomization of the remaining screening failures.

**Figure 1:**
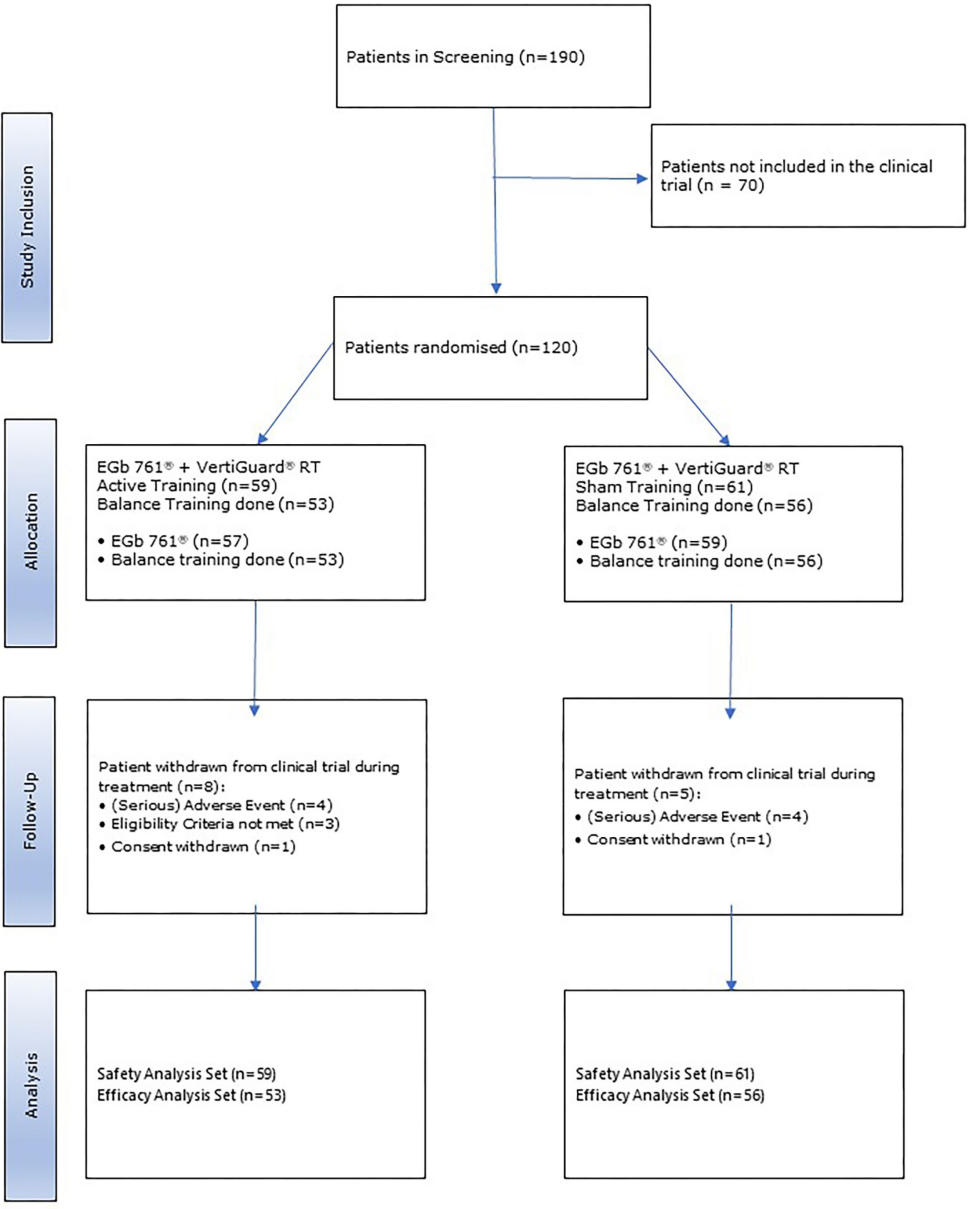
Patient disposition.

Overall, 120 patients received at least one dose of EGb 761. One hundred nine (90.8%) patients received EGb 761 treatment and performed at least one session of balance training. Thereof, 53 patients performed active balance training and 56 performed sham balance training. These patients were analyzed for efficacy (Figure 1).

Thirteen patients discontinued the study prematurely: 8 (13.6%) of the active group and 5 (8.2%) of the sham group. the main reason for premature study discontinuation was adverse events (4 and 4 of the patients, respectively), followed by eligibility criteria not met (3 and 0), and consent withdrawal (1 and 1).

Overall, 67% of the patients were female. The mean age was 74 years, most of the patients were 65–84 years old, mean duration of dizziness was 4.8 ± 4.9 years. Further patient characteristics are summarized in Table 1.

**Table 1:** Patient characteristics.

	**EGb 761 + active training** **(***N*** = 53)**	**EGb 761 + sham training** **(***N*** = 56)**
Age (years); [Min, Median, Max]	74.0 ± 5.6 [63, 75, 86]	74.0 ± 6.6 [61, 76, 89]
Gender (% female)	73.6	60.7
Duration of dizziness (years)	4.1 ± 3.4	5.5 ± 5.9
gSBDT-CS	55.8 ± 10.3	56.3 ± 11.9
DHI sum score	46.5 ± 14.4	41.9 ± 12.7
Functional impact	17.0 ± 6.3	15.7 ± 6.3
Physical impact	16.6 ± 4.6	15.3 ± 5.4
Emotional impact	12.9 ± 6.6	10.9 ± 5.9
Previous non-drug treatments for dizziness (%)		
Any	18.9	23.2
Physiotherapy	3.8	12.5
Physical Exercise	7.5	3.6
TMT A (s)	51.0 ± 15.2	54.2 ± 18.9
TMT B (s)	126.7 ±57.1	119.6 ± 56.1
Mean hearing threshold (dB HL)	33.3 ± 14.7	34.0 ± 14.1
Concomitant medication (%)		
Any	88.7	85.7
agents acting on the RAAS	54.7	44.6
beta blocking agents	28.3	35.7
antithrombotic agents	39.6	21.4
Concomitant diseases (%)		
Any	88.7	85.7
Vascular disorders	64.2	60.7
Metabolism disorders	47.2	39.3
Musculoskeletal disorders	41.5	41.1

**Figure 2:**
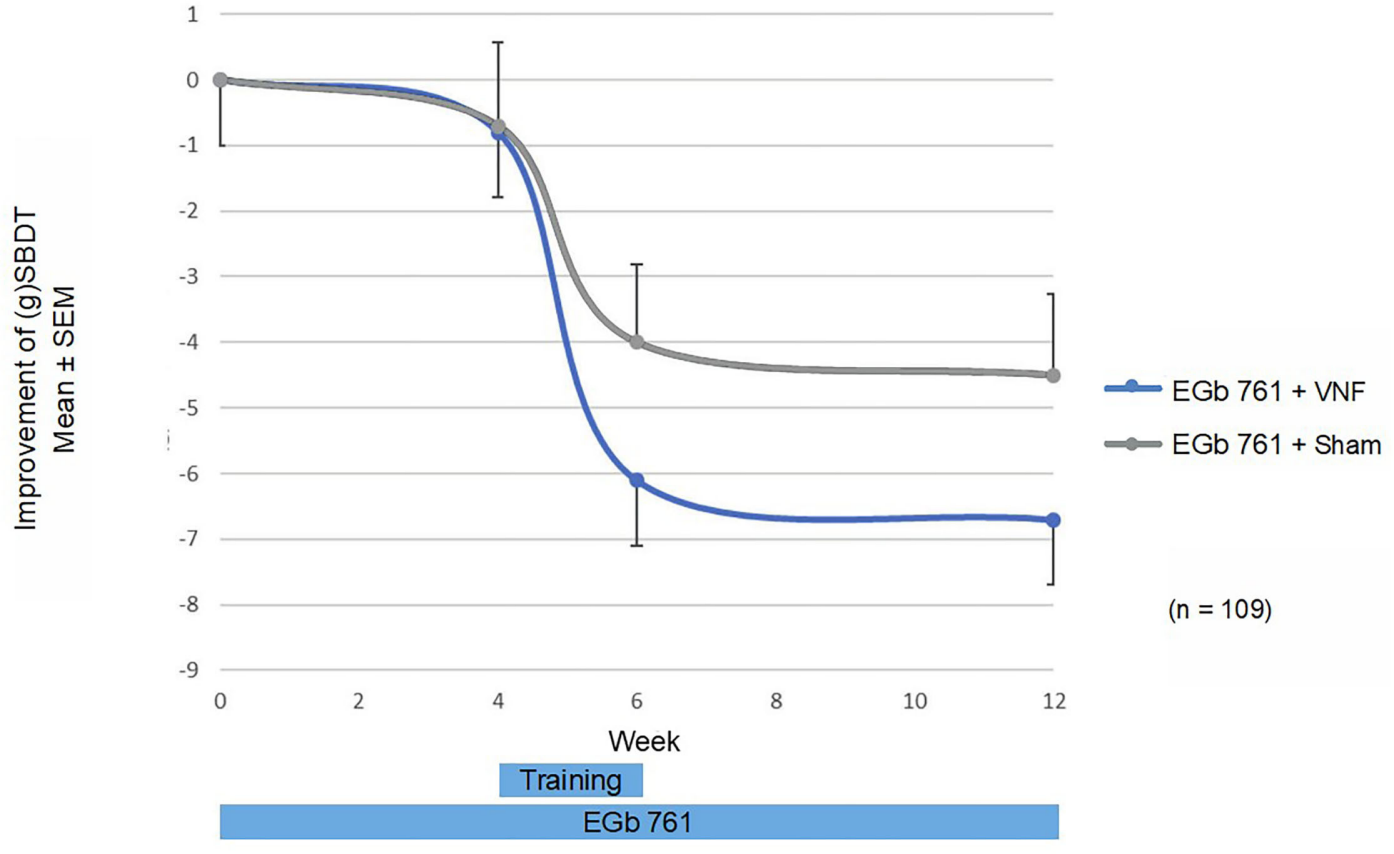
gSBDT composite score: Change from baseline by visit and treatment group.

**Figure 3:**
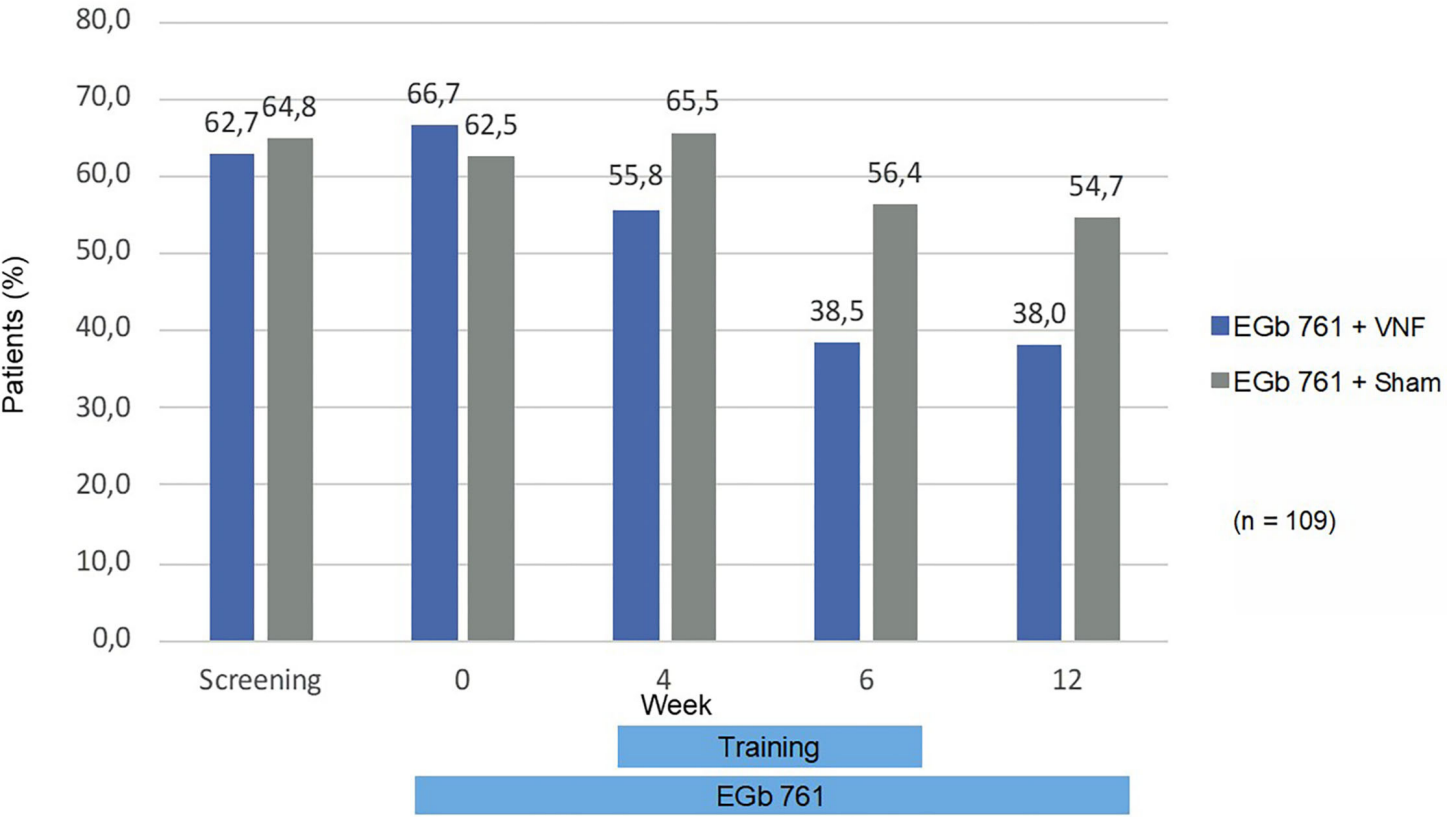
Patients with fall risk >50% (gSBDT-CS). Percentages are based on number of patients that attended to the respective visit and had a not missing value for the gSBDT composite score.

The fall risk in balance-related situations (gSBDT composite score) improved during treatment by 6.7 in the active training group, 95% CI (−9.2; −4.2), and by 4.5 in the sham training group, 95% CI (−7.3; −1.7). The largest and statistically significant improvements were seen in the proprioceptive component [−23.9 (−42.4; −5.4) vs. −10.7 (−25.0; 3.6)], whereas changes in the vestibular [5.5 (−5.1; 16.1) vs. 2.2 (−5.7; 10.0)] and visual component [−2.0 (−10.3; 6.4) vs. −7.4 (−16.5; 1.7)] were smaller and statistically not significant. In the primary endpoint, there was a non-significant difference in favor of the active training group −2.54, 95% CI (−5.4; 0.6), *p* = 0.109 as displayed in Figure 2. Also, in all sensitivity analyses and in secondary analyses of the gSBDT there was a non-significant difference in favor of the active training group. An example is displayed in Figure 3 (patients with fall risk >50%).

**Figure 4:**
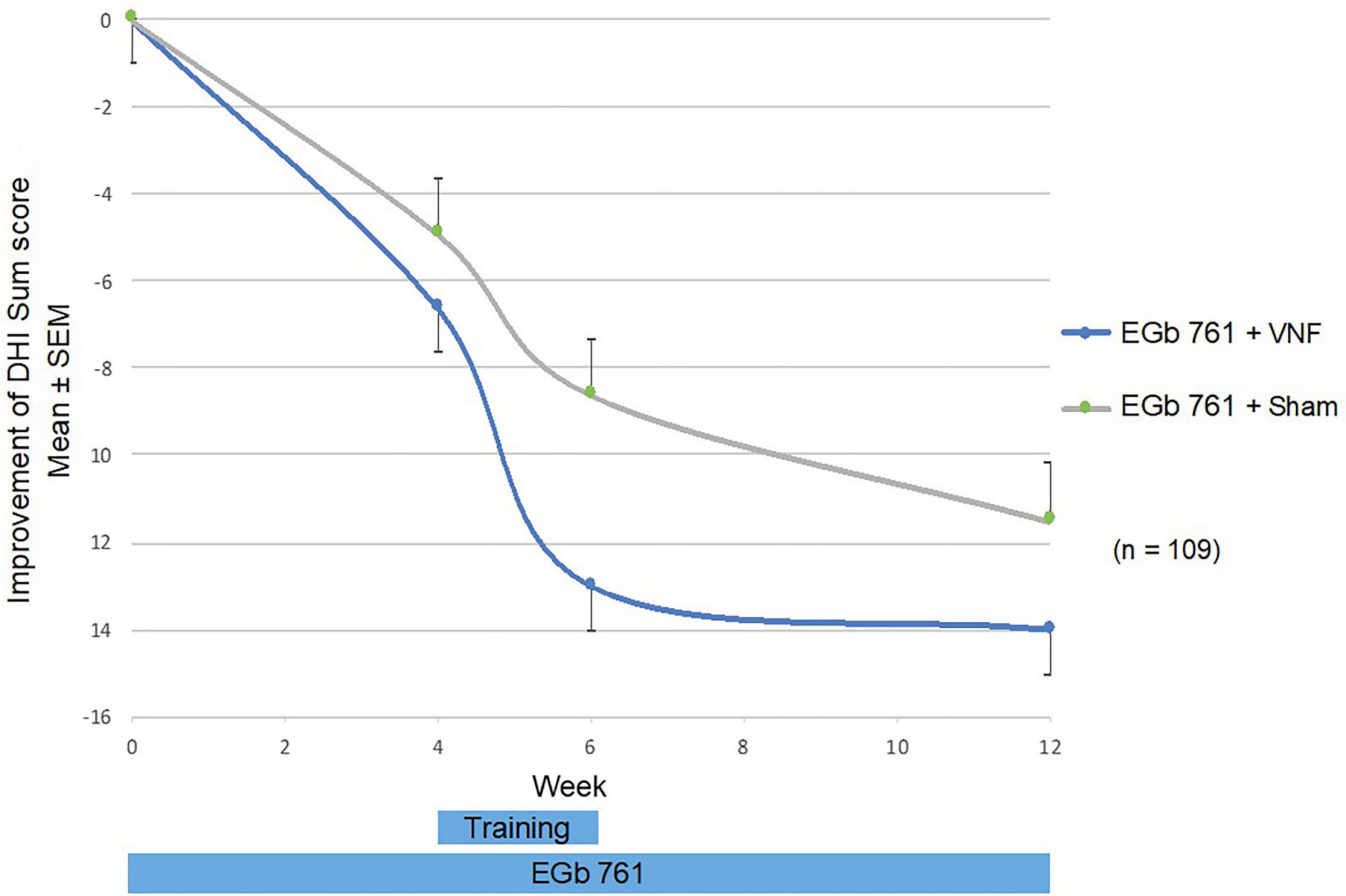
DHI sum score: Change from baseline by visit and treatment group.

The DHI sum score decreased from 44.1 to 31.1 in the total sample (Figure 4) with a treatment group difference after 6 weeks of −3.1, 95% CI (−7.1; 0.9), *p* = 0.132. Most pronounced differences were observed in the physical subscore, i.e., questions related to dizziness aggravated by movements such as walking down a sidewalk or a supermarket aisle or doing homework (Table 2).

**Table 2:** DHI subscores, Change from Baseline at week 6 (MMRM model, estimate ± standard error).

	**EGb 761 +** **active training** **(***N*** = 53)**	**EGb 761 +** **sham training** **(***N*** = 56)**	***P*****-value** **for group** **difference**
Functional impact	−4.2 ± 0.7	−3.9 ± 0.7	0.756
Physical impact	−4.4 ± 0.6	−2.6 ± 0.6	0.041
Emotional impact	−3.6 ± 0.6	−2.6 ± 0.6	0.211

During 12 weeks of treatment with EGb 761, the cognitive performance measured by the Trail Making Test B improved overall (mean) from 123.0 to 102.4 s, 95% CI (−25.9; −12.7). Improvements in hearing ability were small and not statistically significant (between 0 and 2 dB).

In general, the different treatments were well-tolerated. Overall, 26 patients (44.1%) in the active training group experienced 40 adverse events (AE) and 24 patients (39.3%) in the sham group 38 AEs after first drug intake. The most common AEs belonged to the classes infections and infestations, nervous system disorders, gastrointestinal disorders and skin and tissue disorders. Thirty-one of the 78 AEs were considered potentially drug related (relationship “unlikely” or “possible”), no AE was considered device-related by the investigators. Four (*n* = 4) AEs from four (*n* = 4) patients (6.8%) in the active training group and one AE (1.6%) in the sham group were reported as serious adverse events (SAEs). No adverse event leading to death was reported. All SAEs were not related to use of the medical device. One SAE was unlikely to be related to EGb 761, all others were not related. Falls were reported for 4 patients, 2 in each group, leading to hospitalization in one case.

## Discussion

Over many years, balance training (vestibular rehabilitation) has been established as a tool in improving stance and gait stability in the elderly, in our days including internet-based approaches (27). Balance exercises (vestibular rehabilitation) exceed the classical physiotherapy programs [e.g., (28)] in outcome, particularly when a vibrotactile neurofeedback signal is added (9). However, Menant (29) reported in a multifactorial, tailored, single blind, randomized, controlled trial in 392 elderly patients performed in a home setting that such evidence-based therapies reduced the dizziness handicap, but did not improve the physical function of the patients.

This is reasonable insofar as the reduction in muscle mass in the elderly (can be treated by increased protein/meat intake and strength exercises) or the frailty due to osteoporosis (can be treated by vitamin D/calcium intake and increased mobility) are other disease entities of the elderly which are co-factors in the pathogenesis of age-related vertigo and dizziness. Polypharmacy and chronic medical conditions—among others—have been identified as important risk factors for falls and fall-related injuries (30, 31). Our sample was characterized by those multiple comorbidities and the patients took multiple concomitant medications. Therefore, our sample is quite representative of older adults presenting with chronic dizziness.

In this difficult-to-treat population, we observed meaningful improvements in physical function from baseline with EGb 761 and sham training, but even larger improvements with vibrotactile neurofeedback in addition to EGb 761. Because all patients received EGb 761 in this trial, we cannot quantify to what extent EGb 761 contributed to the observed effects.

The results of this trial cannot be readily generalized to treatment with other Ginkgo products. Various preparations made from the leaves of *Ginkgo biloba L*. are available worldwide. Product category (e.g., food supplement, traditional herbal medicinal product, fully approved drug) depends on local legal regulations, resulting in highly variable regulatory and quality requirements. High variability of constituents and even adulterations have been reported for food supplements (32), but differences in pharmacologically active constituents have even been found in fully regulated drugs (33). EGb 761 is a highly standardized product meeting quality requirements for approved medicinal products.

In general, the improvement in the risk of falling (gSBDT composite score) in our trial was comparable to that described for vibrotactile neurofeedback in other studies which included specific vestibular disorders, but also elderly Parkinson's patients (9, 10, 34). In the trial in patients with Parkinson's disease, the SBDT total score improvement by 15 points from a high baseline value of 71.4 was paralleled by a significant reduction of the numbers of falls over 3 months from 18.6 to 6.4 (34). Therefore, we interpret the gSBDT improvement observed in our trial as moderate, but clinically relevant.

Unexpectedly, the sham training group improved too, resulting in a non-significant group difference. In a previous trial, this specific sham training consisting of about 10 min simple balance exercises for 10 days only had no measurable effect (9). Apparently, combining sham training at subthreshold intensity with EGb 761 led to a measurable improvement. This is in line with previous placebo-controlled trials, reporting that EGb 761 enhanced the effects of balance training (12, 13). In various animal models of learning EGb 761 facilitated acquisition of new behavior (35–37). Moreover, the compound accelerated vestibular compensation (38). These effects have been related to radical-scavenging activity, effects on serotonergic neurotransmission and hippocampal glucose metabolism. Moreover, a larger proportion of patients in the sham group had been pretreated with physiotherapy and might therefore have been more responsive to balance training. Finally, studies investigating neurofeedback using a single-blind design consistently resulted in smaller group differences than studies with double-blind design (39). Informing study nurses conducting the training on treatment allocation may cause suggestive effects, which in the end led to the improved effects in the sham training group. The success of patient blinding was not formally assessed. As none of the patients had used the VertiGuard^®^ device before and every patient received vibro-tactile feedback during training, it is unlikely that patients were able to identify whether they received correct or sham feedback.

Inclusion criterion for this trial was an elevated risk-to-fall. In the age group 65+, the annual fall incidence is 12% to 20% (40). In this trial, the observed fall incidence over 12 weeks was 3%. Although fall risk was not an a-priori defined endpoint, this observation might indicate a reduction of fall risk by the treatment administered in this trial. This effect was particularly remarkable after 6 and 12 weeks of intervention, respectively (Figure 3). These improvements were paralleled by a remarkable improvement of cognitive performance during 12 weeks of intake of EGb 761. The observed mean improvement in the TMT B, a measure of executive function, is comparable to improvements reported in patients with mild cognitive impairment (41). In the normative sample for Germany, TMT-B performance declined by 3 s each year of life (42). The observed improvement by 21 points would translate into amelioration of the natural decline over 7 years. Gschwind et al. (43) reported that a standardized *Ginkgo biloba* extract improved dual-task-related gait performance in elderly patients with mild cognitive impairment, specifically an increase of cadence into the normal range and a reduction of stride time variability. Performing more steps per minute led to spending more time in a stable double support phase, thus reducing the fall risk. Moreover, we observed the largest effects of our interventions on the sensomotoric subscore of the gSBDT. Improvement in proprioception is a known effect of vestibular rehabilitation in general (44). Wiesmeier et al. showed that balance training reduced overactive proprioceptive feedback and restored vestibular orientation in the elderly. Chronic treatment with EGb 761 has been reported to alleviate autonomic neuropathy in an animal model of diabetes (45) and peripheral neuropathy in a chronic constriction injury model (46) by scavenging reactive oxygen species. Thus, the radical-scavenging properties of the compound might have contributed to improved peripheral sensomotoric function.

The improvement in the DHI sum score was considerably larger in the active group with VNF plus EGb 761 than previously reported for VNF alone (9) or in combination with cinnarizine plus dimenhydrinate (10) as well as for any other intervention tested in controlled trials in age-related vertigo and dizziness (27–29, 47). Although the mean baseline DHI sum score was higher than in some of these studies, Stam et al. (47) reported a smaller improvement from an even worse baseline with 13 weeks of a multifactorial intervention including exercise therapy. The minimum clinically relevant change in the DHI was identified as 11 points (48), a criterion that was met in the sham training group but was exceeded in the active training group in our trial. Tamber et al. (48) defined the minimally important change as the best cut-off point to discriminate between ‘improved’ and ‘unchanged’ participants on the Disability Scale in a sample of patients with chronic dizziness of mainly vestibular origin. A DHI improvement of 11 or larger indicates, that patients do experience their condition as improved.

A dissociation between self-perception and actual vestibular handicap has been reported earlier, particularly for the elderly (49, 50). The 95% CIs of improvements from baseline in both objective (gSBDT) and subjective (DHI) measures of age-related vertigo and dizziness did not include zero in both treatment groups and in the total sample. This is indicative of significant improvements from baseline with EGb 761 and sham training, but even larger ones with vibrotactile neurofeedback in addition to EGb 761. Although the group differences in the primary endpoint were not statistically significant, the active group consistently showed more pronounced effects in all variants of the primary analysis and in all secondary analyses of gSBDT compared to the sham group. The consistently better results in the active group are supported by the secondary endpoints.

Unspecific effects (e.g., regression to the mean, research participation effect) might have contributed to the improvements observed. In patients seeking medical treatment for vertigo, unspecific effects can be expected to a certain extent in the vertigo-related endpoints. We observed differential improvements in gSBDT components: Statistically significant improvements in the proprioceptive component, but not in the vestibular and visual component. In the DHI, we observed a significant group difference in the physical impact subscore as compared to the functional and emotional impact subscores. The heterogeneity of improvements does not support the interpretation as unspecific only.

Overall, during 12 weeks treatment with individually adapted balance training with vibrotactile neurofeedback and EGb 761 we observed a clinically relevant improvement of the symptoms of age-related vertigo and dizziness with a good safety profile. The data are suggestive of a beneficial effect of the treatment studied but did not conclusively demonstrate such an effect. Confirming the results in future research would represent a remarkable step toward “reducing the symptoms of chronic dizziness and alleviating the resulting physical, psychological, and social disability” in the elderly (51).

## Data Availability Statement

The datasets presented in this article are not readily available because the informed consent received from patients does not cover transfer of raw data to third parties. Requests to access the datasets should be directed to martin.burkart@schwabe-group.com.

## Ethics Statement

The studies involving human participants were reviewed and approved by Ethik-Kommission des Landes Berlin, Fehrbelliner Platz 1, 10707 Berlin, Germany. The patients/participants provided their written informed consent to participate in this study.

## Author Contributions

AE, DB, and MB designed the trial. LD, DB, and AE acquired subjects and data. All authors interpreted the data and prepared and approved the manuscript.

## Funding

This study received funding from Dr. Willmar Schwabe GmbH & Co. KG. The funder had the following involvement with the study: Clinical trial sponsor according to ICH-GCP and DIN EN ISO 14155.

## Conflict of Interest

MB is employed by the company Dr. Willmar Schwabe GmbH & Co. KG. The remaining authors declare that the research was conducted in the absence of any commercial or financial relationships that could be construed as a potential conflict of interest.

## Publisher's Note

All claims expressed in this article are solely those of the authors and do not necessarily represent those of their affiliated organizations, or those of the publisher, the editors and the reviewers. Any product that may be evaluated in this article, or claim that may be made by its manufacturer, is not guaranteed or endorsed by the publisher.

## Abbreviations

AE: Adverse Event
ANCOVA: Analysis of Covariance
CI: Confidence Interval
DHI: Dizziness Handicap Inventory
gSBDT: geriatric Standard Balance Deficit Test
PTA: Pure Tone Audiometry
SAE: Serious Adverse Event
VNF: Vibrotactile Neurofeedback.
